# The recognition and management of neuropsychiatric symptoms in early Alzheimer's disease: a qualitative study among Dutch memory clinic physicians

**DOI:** 10.1111/psyg.12874

**Published:** 2022-07-10

**Authors:** Willem S. Eikelboom, Najoua Lazaar, Rozemarijn L. van Bruchem‐Visser, Francesco U.S. Mattace‐Raso, Michiel Coesmans, Rik Ossenkoppele, Esther van den Berg, Janne M. Papma

**Affiliations:** ^1^ Department of Neurology and Alzheimer Center Erasmus MC Erasmus MC University Medical Center Rotterdam The Netherlands; ^2^ Department of Geriatrics and Alzheimer Center Erasmus MC Erasmus MC University Medical Center Rotterdam The Netherlands; ^3^ Department of Psychiatry Erasmus MC University Medical Center Rotterdam The Netherlands; ^4^ Department of Neurology & Alzheimer Center Amsterdam Amsterdam University Medical Centers Amsterdam The Netherlands; ^5^ Clinical Memory Research Unit Lund University Malmö Sweden

**Keywords:** Alzheimer's disease, behavioural symptoms, dementia, neuropsychiatric symptoms, qualitative research

## Abstract

**Background:**

Timely recognition and treatment of neuropsychiatric symptoms (NPS) in Alzheimer's disease (AD) dementia may improve quality of life, reduce caregiver burden, and delay disease progression. However, management of NPS in early AD dementia remains challenging. To date, little is known about the specific challenges for memory clinic‐based physicians. The aims of this qualitative study were to obtain insights regarding the recognition and treatment of NPS in AD dementia in the memory clinic, to identify challenges experienced by physicians while managing NPS, and to examine the attitudes of memory clinic physicians on the role of the memory clinic in the care for NPS in early AD dementia.

**Methods:**

Semi‐structured interviews were conducted with 13 physicians working at a memory clinic in the Netherlands (*n* = 7 neurologist, *n* = 6 geriatrician, 46% female). The data were analyzed by two independent researchers using thematic analysis.

**Results:**

We observed large variation among Dutch memory clinic physicians regarding care practices, expertise, and attitudes on the role of the memory clinic considering NPS in AD dementia. The most prominent challenges that memory clinic physicians experienced while managing NPS included that the outpatient setting complicates the recognition and treatment of NPS, a lack of experience, knowledge, and/or resources to adequately apply non‐pharmacological interventions, and a lack of consensus among physicians on the role of the memory clinic in NPS recognition and management.

**Conclusions:**

We identified challenges that need to be addressed to improve the early recognition and adequate management of NPS in AD dementia at the memory clinic.

## INTRODUCTION

Neuropsychiatric symptoms (NPS) include a wide range of symptoms including apathy, agitation, affective disturbances, and psychotic symptoms.^1^ NPS are prevalent among individuals with early Alzheimer's disease (AD) dementia,^2^, ^3^ and put a large burden on people living with AD dementia and their caregivers.^4^, ^5^ Furthermore, the presence of NPS is related to an increased risk of incident AD dementia, a faster cognitive decline, and earlier institutionalisation.^6^, ^7^, ^8^

International guidelines recommend non‐pharmacological interventions as first‐line treatment for NPS in dementia.^9^, ^10^ Examples of such non‐pharmacological interventions include caregiver support, psychoeducation, and enhancing tailored activities and these interventions are shown to be effective in reducing NPS.^11^, ^12^ Pharmacological treatments have only limited effect on NPS in early dementia and may lead to serious side effects.^13^, ^14^

Early identification and treatment of NPS seems imperative given the significant impact of NPS on the quality of life of patients and their caregivers,^4^, ^5^ and the associations with accelerated cognitive decline and institutionalisation.^6^, ^7^, ^8^, ^15^ Memory clinics can play a role in the timely care for NPS in early AD dementia, as these multidisciplinary facilities offer a comprehensive diagnostic process and have the potential to offer post‐diagnostic care and support.^16^ In the Netherlands, patients present their cognitive complaints to their general practitioner. After general evaluation, the general practitioner may refer to the memory clinic, which is generally situated as part of the local hospital. General practitioner visits and referral to the memory clinic are covered by mandatory healthcare insurance in the Netherlands, making these facilities highly accessible. At the Dutch memory clinic, a multidisciplinary team that may include neurologists, geriatricians, psychologists, specialised nurses, and psychiatrists usually provide a standardised diagnostic work‐up consisting of medical history taking, neurologic examination, neuropsychological assessment, laboratory testing, and neuroimaging.^17^

NPS are often underdiagnosed during the diagnostic stage of AD dementia and effective non‐pharmacological interventions are hardly implemented in the care for NPS in individuals with AD dementia at the memory clinic.^18^ Previous studies have identified several factors that contribute to the complexity of care for NPS, including the multifactorial cause of NPS,^19^ their fluctuating nature,^2^, ^20^ and difficulties in distinguishing NPS in dementia from primary psychiatric disorders.^21^, ^22^ Furthermore, the fact that the diagnosis of AD dementia strongly relies on cognitive and functional deficits further hampers the recognition of NPS in early AD dementia.^23^

In addition to the challenges mentioned above, there may be factors related to the specific care setting contributing to the complexity of care for NPS in AD dementia. Several qualitative studies among nursing home staff and general practitioners have indeed revealed unique challenges for these specific care settings, such as a perceived lack of time among nursing home staff and general practitioners, conflicting expectations on the treatment plan between general practitioners and family members of patients, and a perceived priority of care tasks over personal interaction among nursing home staff.^24^, ^25^, ^26^ In addition to physicians working in primary care and nursing homes, we have indications that physicians also experience difficulties with assessing and managing NPS in the memory clinic setting.^22^, ^27^However, there is a lack of knowledge on the current care for NPS in early AD dementia at the memory clinic and what kind of challenges physicians face in the care for NPS in AD within this specific setting.

A better understanding of the experiences and attitudes of memory clinic physicians on the current care for NPS is necessary to identify challenges that need to be overcome to improve timely diagnosis and treatment of NPS in early AD dementia. Therefore, the aims of the current study were to: (i) obtain insight in the current assessment and management of NPS in early AD dementia in the memory clinic; (ii) identify challenges experienced by physicians while managing NPS in early AD dementia in this specific care setting; and (iii) examine attitudes of memory clinic physicians on the role of the memory clinic in the care for NPS in early AD dementia.

## METHODS

Ethics approval was granted by the Medical Ethics Committee Erasmus MC of Rotterdam, the Netherlands (MEC‐2020‐0249). All participants gave informed consent. The Standards for Reporting Qualitative Research were followed for reporting this qualitative study.^28^

### Sampling and recruitment

We included neurologists and geriatricians working at a memory clinic who regularly diagnose and treat patients with early AD dementia. In the Netherlands, memory clinics are primarily coordinated by geriatricians and neurologists.^17^ These medical specialists are responsible for the diagnosis and treatment of patients, while referring to psychologists, psychiatrists, nurses, and/or social workers for additional diagnostic purposes or support. Therefore, we only included neurologists and geriatricians in this study. Participants were recruited via two strategies. A part of the participating physicians were already involved in an intervention study to improve the management of NPS in AD dementia in the memory clinic as part of the BEAT‐IT project.^29^ These physicians were interviewed during the first observational wave of the project, in which patients received care as usual and served as a control group (convenience sample). Furthermore, additional physicians were contacted to ensure maximum variation regarding profession (neurologist/geriatrician), type of hospital where they are employed (general/academic), and years of experience (purposive sampling). We continued inclusion until saturation was achieved.^30^

### Semi‐structured interview

One researcher (W.S.E.) conducted the interviews either face‐to‐face or via telephone due to COVID‐19 restrictions. All interviews were audio‐taped after obtaining verbal informed consent.

The topic guide was developed prior to the start of the first interview, but was adapted halfway based on consensus among the researchers (see Table 1 in Supporting Information). The semi‐structured interviews revolved around the following topics: experiences of memory clinic physicians when managing NPS in early AD dementia; challenges they encounter in their daily clinical practice considering the management of NPS; attitudes on who is responsible for the care for NPS in community‐dwelling patients with early AD dementia; and perspectives on the ideal care for NPS. Questions were asked in an open non‐directive manner focusing on the participants' attitudes and experiences. Each question was explicitly related to AD to ensure that physicians addressed AD in their responses. When in doubt, the interviewer asked specifically whether the response was related to individuals with AD. Physicians were encouraged to discuss examples of cases they encountered in their daily clinical practice.

### Analysis

**Figure 1:**
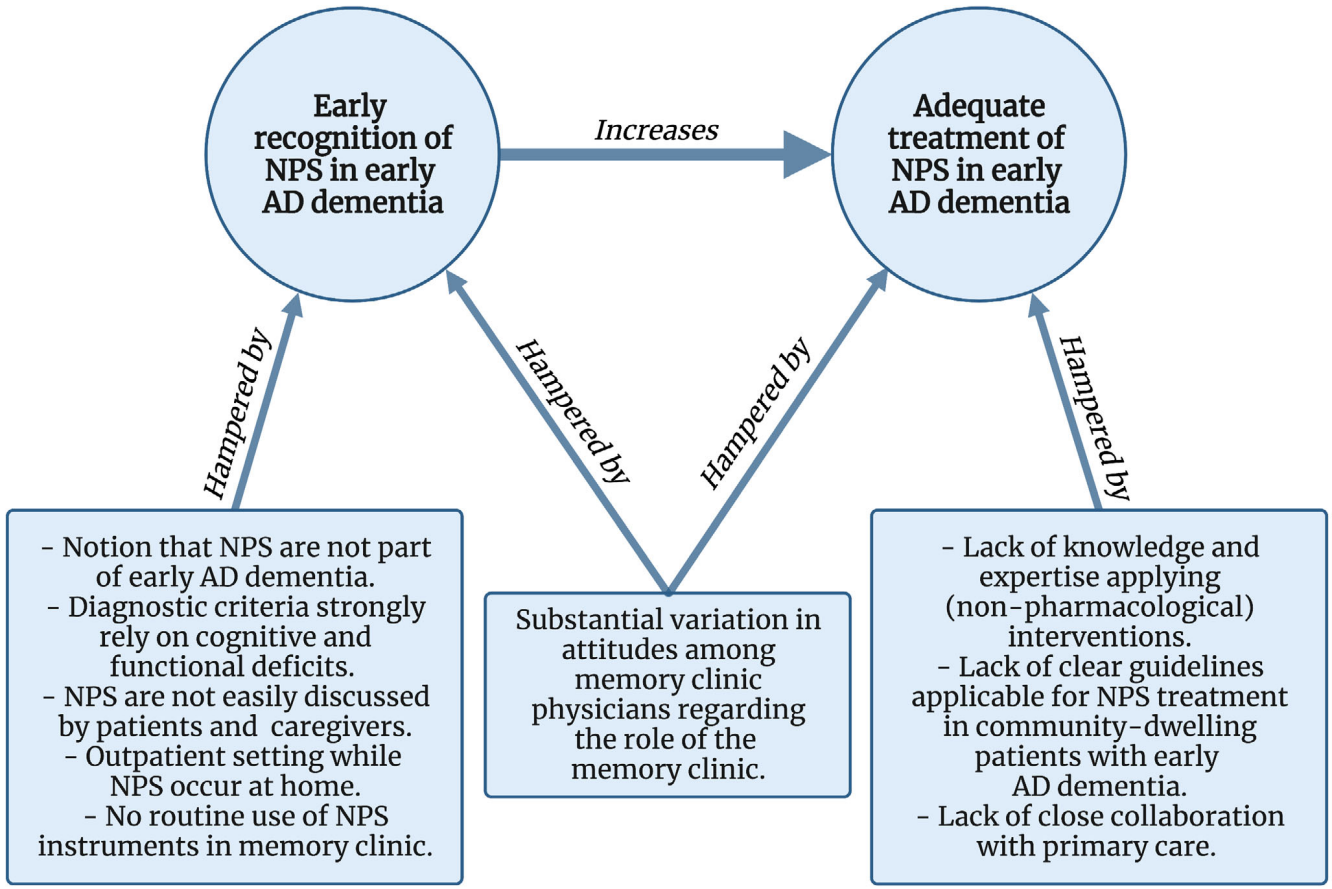
The challenges identified that hamper early recognition and adequate treatment of NPS in early AD dementia. *Notes*. AD, Alzheimer's disease; NPS, neuropsychiatric symptoms.

The audiotapes of all interviews were transcribed verbatim and de‐identified prior to data analysis. The data were analysed by two independent researchers (W.S.E. and N.L.) following a thematic analysis approach.^31^ The coding and analyses were an iterative process in parallel with the interviews, allowing for adjustment of questions and topics. After familiarising with the data, both researchers proposed a code book consisting of open codes that emerged from the data. These code books were discussed resulting in a final code book used to systematically code the data. The final coding consisted of open coding followed by axial coding and selective coding. Next, both researchers independently collated the codes into preliminary categories and themes. Finally, initial themes were redefined through discussion between all researchers resulting in the three key themes: (i) recognition of NPS; (ii) management of NPS; and (iii) role of the memory clinic in care for NPS in early AD dementia (Fig. 1).

## RESULTS

Thirteen of the 14 physicians that were invited to be interviewed agreed to participate. One geriatrician declined to participate because of a lack of time due to additional COVID‐19 care. Characteristics of the participants can be found in Table 1. Although physicians with a background in neurology and geriatrics both had experience ranging from <10 years to >20 years, neurologists had more years of experience in the memory clinic (median [range] = 12.0 years [8.0‐30.0]) than geriatricians (7.0 years [4.0‐21.0]).

**Table 1:** Characteristics of the 13 memory clinic physicians included in this study

	*n* (%)
Gender	
Female	6 (46.2%)
Male	7 (53.8%)
Profession	
Neurologist	7 (53.8%)
Geriatrician	6 (46.2%)
Type of hospital employed	
General	8 (61.5%)
Academic	5 (38.5%)
Years of experience in the memory clinic	
<10 years	6 (46.2%)
10–20 years	4 (30.7%)
>20 years	3 (23.1%)

### Recognition of NPS

Most memory clinic physicians (*n* = 10/13) indicated they frequently detect NPS such as apathy, irritability, depression, and anxiety in the patients they diagnose and treat with early AD dementia. Half of the physicians indicated they always address NPS as part of their standard diagnostic work‐up and they repeatedly emphasised the importance of these symptoms:

*‘I find it hard to imagine that you don't pay attention to NPS, because I think that this is something that caregivers struggle with the most. It is always about the behaviour.’ (Neurologist #7)*

A neurologist suggested that not all physicians are aware that NPS are part of dementia and that therefore more education is needed:

*‘I think it's needed to highlight more often that dementia is more than cognitive impairment during our residency programs. And also to stress that especially these behavioural problems, these neuropsychiatric symptoms lead to major burden in patients, but also among family members.’ (Neurologist #6)*

Three physicians included in our study considered NPS not as prominent symptoms in individuals in the early phase of AD dementia who they encounter at the memory clinic:

*‘I don't see that [NPS] much in the beginning of the disease, but later on in almost all patients. As the disease progresses, you see more of these symptoms.’ (Neurologist #4)*

These physicians reported that the evaluation of NPS is not part of their standard diagnostic work‐up as their focus is on cognitive functioning to establish a dementia diagnosis.

There was consensus among the participants who frequently detect NPS that physicians need to actively address NPS in order to evaluate its presence and clinical relevance. Physicians suggested that patients and caregivers may feel hesitant to bring up NPS because of feelings of shame, difficulties describing these symptoms, and to avoid making the patient upset or angry. Furthermore, one neurologist pointed out that physicians may feel hesitant to address NPS as well because they have no subsequent strategy for managing these symptoms:

*‘I'm usually not inquiring about NPS as it is hard to treat, because you don't have a solution immediately. So, although I may detect it, I don't have a tailor‐made solution ready.’ (Neurologist #4)*

Many physicians described that the setting of the outpatient memory clinic makes it difficult to recognise NPS as these symptoms mostly occur at home:

*‘The hardest of all with NPS observed at an outpatient clinic is that you see patients for only a very short period of time and within a very specific setting, although the problems arise very often within the interaction between patient and caregiver. (…) This setting is just not suited for finding a solution for NPS that occur at home.’ (Geriatrician #3)*

However, several physicians also gave examples of NPS that they observe when patients and caregivers visit the memory clinic together:

*‘The benefit of having both patient and caregiver in the doctor's office is that one can observe what also occurs at home. For example, if a patient says: ‘that is totally untrue what you are saying.’ and if the caregiver then also reacts in an agitated manner, I usually explain how the caregiver could better deal with this.’ (Neurologist #1)*

Half of the physicians worked at a memory clinic in which NPS assessment scales such as the Neuropsychiatric Inventory (NPI) and the Geriatric Depression Scale (GDS) are part of the standard diagnostic work‐up. However, only one physician mentioned using these scales to guide the diagnosis of NPS in AD at the memory clinic. Another physician argued that merely using standardised screening tools might not be sufficient to fully capture NPS and may hamper an adequate response:

*‘I don't believe in this [using checklists to screen for NPS]. One should have a conversation with people and during this conversation, one should address these symptoms systematically. But just checking off these symptoms for the sake of it results in an awkward conversation that does not provide the correct information needed.’ (Neurologist #6)*

### Management of NPS

Half of the group of physicians were aware of the existing Dutch guidelines for the treatment of NPS in dementia, but only one physician uses these guidelines regularly in daily clinical practice. Alternatively, physicians base their treatment on own clinical experience, peer consultation, literature research, and research findings presented at national conferences. One geriatrician acknowledged that the current guidelines for NPS are difficult to apply in the memory clinic setting:

*‘It makes it in particular difficult to use, because these guidelines for NPS are originated at the nursing home setting in which non‐pharmacological interventions have way more potential benefit.’ (Geriatrician #4)*

Several physicians described that they sometimes experience a tension between the distress associated with NPS among caregivers and a lack of awareness of the presence of NPS and associated distress among patients. Two physicians experienced this even as an ethical dilemma as they wondered whether they should treat patients who do not experience any burden, while their caregivers do report severe NPS that causes substantial distress:

*‘Should one act if a patient who you are treating does not have any complaints, but the caregiver who you are not formally treating does have serious complaints? But caregivers are essential, so if they are in distress and experience severe burden, one should do something with these complaints right?’ (Geriatrician #4)*

*‘You should always be aware that you are treating the patient and not the caregivers. I don't think you should treat a patient with medications in order to comfort caregivers. The patient should benefit from this too.’ (Geriatrician #3)*

Physicians differed substantially in the amount of experience they have with managing NPS and whether they feel competent while doing so. This was unrelated to the number of years that they worked at the memory clinic. The vast majority of the physicians treat NPS on a regular basis, while only three physicians indicated they almost never treat NPS. Regardless of how often physicians treated NPS, many stated they experience the care for NPS in early AD as challenging. Two physicians acknowledged they lack specific knowledge considering NPS treatment:

*‘I think that it is also a lack of knowledge on how to deal with these symptoms and how to educate dyads on how to handle this.’ (Neurologist #1)*

Other physicians expressed that they have sufficient experience and expertise to manage NPS, but described other challenges:

*‘I don't find the symptoms in itself particularly difficult to manage, because I see that, when it works out well to really change things, patients and their caregivers are more relaxed. But it's really hard to get other care professionals involved and to create a treatment plan together. So it's more an organisational challenge than the symptoms per se.’ (Geriatrician #6)*

#### Non‐pharmacological interventions

The majority of the memory clinic physicians (*n* = 10/13) preferred non‐pharmacological approaches over pharmacological interventions to treat NPS, especially for specific symptoms including apathy, agitation, and sleep disturbances:

*‘I would say: ‘The doctor as a medicine’, because you don't have much more to rely on. So you have to explain and discuss it.’ (Neurologist #4)*

Although non‐pharmacological approaches were often mentioned and generally preferred over pharmacological interventions, a third of the physicians could not name specific non‐pharmacological interventions and indicated that they rarely apply non‐pharmacological interventions themselves:

*‘It's fine with me to be responsible for pharmacological treatments, but I think that supporting patients and caregivers to deal with these symptoms should take place in the community. (…). Cognitive behavioural therapy maybe? I don't have any experience with that and don't think that I would be able to provide that.’ (Neurologist #3)*

These physicians also expressed the need for additional registered nurses at the memory clinic to support with non‐pharmacological approaches:

*‘In an ideal world, I would like to have a registered nurse who has an appointment with the patient prior to my appointment. (…) Who also pays attention to non‐cognitive complaints, non‐pharmacological intervention and coaching in a way that the medical doctor has more time for the more persistent symptoms that may need a pharmacological treatment.’ (Neurologist #2)*

Two‐third of the physicians indicated that they regularly apply non‐pharmacological strategies including the investigation of underlying triggers and causes, providing patients and caregivers psychoeducation, increasing meaningful activities, referring to a day care centre, giving caregiver support, or enhancing physical exercise. The various psychosocial causes of NPS formed the main reason for physicians to apply non‐pharmacological approaches to manage these symptoms. Examples that were described included a lack of knowledge among caregivers, caregiver burden, pre‐existing personality traits of patients, difficulties coping with a dementia diagnosis, and negative communication styles among caregivers. As one geriatrician illustrated:

*‘Verbal or physical aggression often arises from the interaction between individuals. Paying attention to this really helps to remove the trigger and prevent further escalation.’ (Geriatrician #1)*

#### Pharmacological interventions

All physicians gave examples of patients they treated with psychotropic medications who exhibited very severe and/or persistent NPS that were very distressing for caregivers, caused harm, or hampered home care or other forms of health care. Furthermore, most physicians (*n* = 11/13) felt competent and had experience with treating psychotic symptoms using pharmacological interventions. Yet, for other symptoms such as depression and anxiety, several physicians (*n* = 3/13) mentioned they felt less competent or had insufficient experience to use psychotropic drugs:

*‘We have less experience with the remaining [NPS]. Depressive disorders and anxiety disorders are treated by the psychiatrist (…) as we do this less often, so we don't recognise the side effects of these medications.’ (Neurologist #2)*

All physicians were aware of the limited effectiveness and associated negative side effects of pharmacological treatments when used to treat NPS. Several physicians (*n* = 4/13) mentioned that concerns about the efficacy of pharmacological treatments for NPS and increased risk of serious side effects made them use non‐pharmacological approaches for NPS instead:

*‘In general, I'm very hesitant with pharmacological interventions because you will have side effects very quickly and the effectiveness is questionable at best.’ (Neurologist #6)*

Almost all physicians stated they prescribe psychotropic drugs to treat NPS, although large differences existed in how often physicians do so, with only a minority of physicians who prescribe very commonly. These physicians indicated they sometimes feel powerless while managing NPS as they did not have many alternative treatments available:

*‘I think that it really doesn't matter much… (…) Nothing is safe or effective. It's really a matter of trial‐and‐error.’ (Geriatrician #2)*

*‘Every time that I'm attending a symposium or conferences on NPS, the conclusion is that nothing is effective. That is really demotivating.’ (Geriatrician #5)*

Two physicians expressed they or their colleagues sometimes use pharmacological interventions because they lack the knowledge and experience with using non‐pharmacological approaches:

*‘I think that, because one lacks knowledge [about non‐pharmacological interventions] (…), one is also more inclined to use medications as a medical doctor. (…) You are just more likely to use medications if it's not going well at home, because it has to go well at home otherwise you have a problem.’ (Neurologist #1)*.

In addition, pharmacological treatments were considered less time‐consuming and part of care medical doctors are supposed to provide in a hospital setting:

*‘We, as medical doctors, sometimes have the tendency to ‘think’ solely in terms of pharmacological treatment options instead of non‐pharmacological approaches. Everyone does consider non‐pharmacological interventions as important, but I think that some medical doctors are just used to prescribe medications very quickly in clinical practice. (…) It's just so easy right?! Just one pill, that's all! (…) Maybe it's also because physicians feel that it's supposed to be that way in the hospital?’ (Neurologist #2)*

### The role of the memory clinic in the care for NPS

There was a substantial variation in the attitudes among physicians on the role of the memory clinic in the care for NPS in early AD. Several physicians (*n* = 5/13) argued that the care for NPS belongs predominantly in the primary care setting, while memory clinics should purely focus on establishing a dementia diagnosis. These physicians stated that care provided in memory clinics is too expensive or that there is a risk of medicalisation if community‐dwelling patients and caregivers regularly have to visit the memory clinic. Furthermore, physicians mentioned that in contrast to memory clinic physicians, primary care physicians such as general practitioners and community nurses commonly conduct home visits that enables them to observe NPS in the context in which they occur and can therefore directly intervene. Furthermore, some physicians suggested that clinicians working in other care settings are more experienced in managing NPS:

*‘I don't think that the memory clinic setting is suited to follow up on these kinds of issues. (…) I do think that the diagnostics belongs to us, but it's pretty much completed after that as we don't have anything more to offer. So then it's a kind of waste to keep following these patients within this highly specialised outpatient clinic. I think, in general, that others have more experience with these issues. For example, case managers or community mental healthcare services.’ (Neurologist #5)*

In contrast, other physicians (*n* = 6/13) felt that memory clinics should be actively involved in care for NPS in early AD. Some of them suggested the memory clinic should limit this role to the detection and diagnosis of NPS, whereas others also expressed that the memory clinic should also be involved in the treatment of NPS.

*‘I don't think that the care for NPS should be primarily embedded within the primary care. (…) You don't only look at a diagnosis, but also at everything that comes along with that. (…) I can hardly imagine that you only focus on a part of dementia and leave the rest of it to others. That's just very hard to understand for me.’ (Geriatrician #4)*

Furthermore, two physicians expressed that the NPS diagnosis and treatment do not have to take place at the memory clinic, but that the memory clinic should play an active and coordinating role to ensure that at least some care provider is taking care of NPS:

*‘I would like to see memory clinics play a more active and coordinating role in the care [for NPS] at home, because I see dementia as a terminal illness that deserves excellent care. Imagine that we would to this to patients with cancer…’ (Geriatrician #6)*

Although many physicians acknowledged there are significant regional differences within the Netherlands in how the care for NPS in early AD dementia is organised, there was consensus among memory clinic physicians that the collaboration with primary care providers should be improved. Yet, many physicians mentioned that they experience that at least a part of the general practitioners and case managers they collaborate with lack knowledge and experience concerning both detecting and treating NPS. For some memory clinic physicians (*n* = 3/13), this is a reason why they remain actively involved in the care for NPS.

We found no substantial difference between geriatricians and neurologists regarding care practice and attitudes on the role of the memory clinic. The only remarkable difference was found relating to the time available to address NPS. While the majority of the geriatricians reported they feel they have more time to adequately address NPS compared to general practitioners, the majority of neurologists indicated they experience a lack of time to adequately manage NPS.

## DISCUSSION

This study examined the current state of care for NPS in early AD dementia at the memory clinic and the challenges physicians experience during the assessment and management of these symptoms. We observed substantial variation in the experiences, expertise, and attitudes of physicians working at the memory clinic related to the care for NPS in early AD dementia. Moreover, we identified several challenges that memory clinic physicians experience while managing NPS including the memory clinic setting that makes it difficult to diagnose NPS, a lack of experience, knowledge, and/or resources to adequately apply non‐pharmacological interventions, and a lack of consensus among physicians on the role of the memory clinic in care for NPS.

The majority of the physicians reported they frequently observe NPS in individuals with AD dementia visiting the memory clinic, which is in line with prior studies showing that >85% of the individuals with AD dementia visiting the memory clinic exhibit at least one NPS according to standardised assessment scales such as the NPI.^2^, ^3^ Despite the high prevalence rates of NPS in early AD dementia, these symptoms are not always detected during the diagnostic stage of AD dementia.^18^, ^27^, ^32^ We identified several challenges that physicians experience when assessing NPS that may contribute to the underdiagnosis of NPS in early AD dementia. First, a minority of physicians stated they do not consider NPS as a prominent symptom in the early phase of AD dementia, a view that is commonly shared among clinicians in dementia care.^1^ It is important to make physicians aware of the fact that NPS occur frequently in early AD dementia, even as the first manifestation of the disease.^22^ A good example of such an effort is the development of the concept of mild behavioural impairment, classifying individuals with NPS in the context of no or only mild cognitive impairment who are at risk for developing dementia.^33^ Second, the majority of memory clinic physicians mentioned that the outpatient memory clinic is a difficult setting to detect NPS in early AD dementia as most of these symptoms occur at home. Therefore, physicians have to rely on retrospective information provided by patients and their caregivers to diagnose NPS, instead of witnessing it as it occurs. This results in a third challenge as physicians indicated they find it challenging that they have to rely on information provided by patients and their caregivers as patients and their caregivers do not always report NPS due to feelings of shame, difficulties describing NPS compared to cognitive symptoms, and because caregivers may try to avoid confronting patients with these symptoms. Results extend previous studies that have identified factors that hamper the assessment of NPS based on caregiver estimations such as that caregivers are often initially unaware that NPS are part of the disease,^34^ and caregivers use different terminologies to describe NPS compared to physicians.^35^ Altogether, these factors contribute to the observation that patients and caregivers may have difficulties bringing NPS up in the doctor's office and that physicians need to address and explain these symptoms. The majority of the physicians indicated the need for proactive screening of NPS in order to evaluate its presence and clinical relevance and half of the physicians indicated that NPS scales are part of the standard diagnostic work‐up at their memory clinic, which is in line with a survey among Dutch memory clinics.^17^ However, very few physicians reported they used information from NPS scales to guide the assessment of NPS in AD, highlighting that physicians fail to prioritise the standardised assessment of NPS. This has been reported previously and may hamper the early detection of NPS in AD dementia.^36^

The majority of the physicians indicated the effectiveness of non‐pharmacological interventions over pharmacological treatments. Yet, we observed considerable differences among physicians in the amount of experience and expertise they have in applying non‐pharmacological interventions for NPS in AD dementia. Despite these differences, all physicians indicated they find the use of non‐pharmacological treatments for NPS challenging. Although there is an overall increase in the routine use of psychosocial interventions over the last decades at memory clinics in the Netherlands,^17^ some physicians in our study indicated they rarely applied non‐pharmacological interventions. These physicians reported they lack specific knowledge and do not feel confident to apply these interventions. This has also been reported among general practitioners and nursing home staff.^25^, ^26^ A lack of experience and knowledge on the non‐pharmacological treatment of NPS in AD dementia has serious consequences as our findings show that this can: (i) lead to an underdiagnosis of NPS since physicians feel hesitant to address these symptoms and this (ii) facilitates an increase in the prescription of psychotropic drugs. The physicians who do regularly apply non‐pharmacological interventions reported there is a lack of close collaboration with primary care providers and they sometimes lack sufficient time to assess NPS, examine underlying causes, and follow‐up on treatment advice.

Our findings reveal a lack of consensus among physicians included in our sample on the role of the memory clinic in the care for NPS in early AD dementia. While some physicians argued that the care for NPS should primarily take place in primary care, several others plead that the memory clinic should participate in the care for NPS in AD dementia. This lack of consensus clearly hampers the standardisation of care for NPS in AD dementia. Therefore, it is important that memory clinics need to reach consensus on their role in the care for NPS in AD dementia in order to make clear who is responsible for the diagnosis and treatment of these distressing symptoms, at least at a regional level. Important to note, although several physicians in our study claimed that the care for NPS in AD dementia should be organised in primary care, previous studies have shown that primary care providers such as general practitioners and homecare staff are hesitant or inexperienced to apply non‐pharmacological interventions and also do not always consider this their role.^26^, ^37^ So it should be important to include primary care providers in this discussion as well.

## STRENGTHS AND LIMITATIONS

The mixture of physicians in terms of gender, profession, years of experience, and hospital type is a strength of this study. Moreover, participants were included using both convenience sampling and purposive sampling and analyses were conducted in duplicate to increase validity and generalisability of our findings. However, although no new themes emerged during the final two interviews, the small number of physicians interviewed is a limitation of this study. In addition, we invited neurologists and geriatricians to participate in this study as these medical specialties coordinate the care provided at the memory clinic in the Netherlands, while care professionals such as psychiatrists are only consulted if needed.^17^ However, psychiatrists are commonly part of the standard care provided at memory clinics in other countries.^38^, ^39^, ^40^ Therefore, future studies in other countries are needed to study whether our findings also generalise to memory clinics worldwide. Furthermore, memory clinic physicians were interviewed about their attitudes on the role of the memory clinic in the care for NPS in early AD dementia and about their experiences with other care provides such as general practitioners. Yet, these care providers were not included in this study and future studies are needed to identify the attitudes and needs of primary care providers considering the care for NPS in early AD dementia.

## CONCLUSION

Our results show large variation among memory clinic physicians regarding their care practices, knowledge, and attitudes on the role of the memory clinic relating to NPS in AD dementia. Hereby, our findings help to clarify the discrepancy between the recommendations of international guidelines and daily clinical practice observed in memory clinics. By doing so, we identified challenges that need to be addressed to improve the early recognition and adequate treatment of NPS in the early stages of AD dementia.

## AUTHOR CONTRIBUTIONS

W.S. Eikelboom designed the study, conducted the interviews, analyzed the data, and wrote the article. N. Lazaar analyzed the data and assisted with writing the article. R.L. van Bruchem‐Visser assisted in creating the topic list and assisted with writing the article. F.U.S. Mattace‐Raso assisted with writing the article. M. Coesmans assisted with writing the article. R. Ossenkoppele assisted with writing the article. E. van den Berg designed the study and assisted with writing the article. J.M. Papma designed the study, supervised the study, and assisted with writing the article.

## DISCLOSURE

The authors have no potential conflicts of interests to disclose.

## Supporting information


**Table S1**: Topic list used to guide interviews.

## Data Availability

The data that support the findings of this study are available on request from the corresponding author. The data are not publicly available due to privacy or ethical restrictions.
