# Supplementary material for: The recognition and management of neuropsychiatric symptoms in early Alzheimer's disease: a qualitative study among Dutch memory clinic physicians
Source: Psychogeriatrics. 2022 Jul 10;22(5):707–17. doi: 10.1111/psyg.12874 (PMC9541792; doi:10.1111/psyg.12874)
Supplement: Supplementary file 1 — Table S1: Topic list used to guide interviews. [file PSYG-22-707-s001.docx]

**Table S1.** Topic list used to guide interviews

| **Topic** | **Example questions** |
| --- | --- |
| *Prevalence of NPS* | - How often do you observe NPS in the patient you diagnose with early AD dementia at the memory clinic? - Which specific NPS do you see in the early phase of AD dementia? - Can you give an example of NPS you detected in a patient you recently diagnosed with AD dementia? |
| *Diagnosis of NPS* | - How do you diagnose NPS in AD dementia? - What makes it hard to diagnose NPS within the memory clinic setting? |
| *Management of NPS* | - Some physicians find it hard to manage NPS in early AD dementia, what is your experience? - What makes it hard to treat NPS within the memory clinic setting? - Is your treatment similar for each NPS or do you treat specific NPS differently? If so, why? - What do you know about the use of psychotropic drugs for NPS in early AD dementia? |
| *Use of guidelines* | - Are you familiar with existing guidelines for the diagnosis and treatment of NPS in early AD dementia? - Do you regularly use guidelines to guide diagnosis and treatment of NPS in early AD dementia? - To which extent are current guidelines applicable to use in the memory clinic setting? |
| *Care within the memory clinic setting* | - Do you think that it is important to pay attention to NPS in the memory clinic? - How does the care for NPS in early AD dementia looks like in the ideal world? - To which extent should the memory clinic be involved in the diagnosis and treatment of NPS in early AD dementia? |

*Abbreviations. ad = Alzheimer’s disease, nps = neuropsychiatric symptoms.*
